# Medicine, Pathology and Therapeutics

**Published:** 1899-08

**Authors:** Albert T. Lytle

**Affiliations:** Buffalo, N. Y.


### MEDICINE, PATHOLOGY AND THERAPEUTICS.

Conducted by ALBERT T. LYTLE, M. D., Buffalo, N. Y.

### AUTOINTOXICATION IN EPILEPSY.

WEBER, L. W., (*Münch. Med. Woch.*, June 28, 1898,) in a paper on The significance of autointoxication in epilepsy, says that post mortem findings in cases dying in status epilepticus shows degeneration of the kidneys, the liver and the heart, traces of new or old inflammation in the vessels, especially of the brain and often extravasations of blood in the brain. The clinical manifestations of these cases give indications of poisoning. He believes that there is a large excess of the products of metabolism, especially of the incomplete kinds in this disease, that the individuals afflicted are predisposed either by heredity or by acquired conditions, that treatment should be eliminative and that bromides should be a last resort.

---

### WARBURG'S TINCTURE.

VAN MARTER, J. G., JR., of Savannah, Ga., (*Therapeutic Notes*) in a paper read before the clinical society of St. James's Dispensary, says: "Warburg's tincture should always be given as recommended by experienced practitioners in India, after a brisk purge, undiluted, in doses of half ounce, all drinks withheld, repeated in three hours, and the patient carefully rolled up in blankets to encourage the profuse aromatic perspiration which follows. It is one of the most powerful diaphoretics known; it is also a diuretic, a stimulant, and a purgative. I always follow its use by opium and small doses of whisky—at least never omit the opium, which I believe acts most happily."

NUCLEIN IN THE TREATMENT OF SEPTICEMIA.

COURTNEY, WALTER, (*Medical News; Therapeutic Notes*) has tried nuclein in the treatment of patients suffering from septicemia, and the results, as shown in ten cases, were very satisfactory, only one proving fatal. He sums up his experience thus: (1) The yeast nucleinic acid should be given as soon as septic infection is suspected. Do not wait for the typical, classical symptoms as given by text-books. If delay occurs until all the symptoms of the disease are added to an abnormal increase in temperature and pulse-rate, a serious waste of most valuable time must occur; (2) the nuclein should always, in septicemia at least, be given hypodermatically if possible, in order to secure prompt action. If the 1 per cent. solution is used, at least from thirty to forty minims, undiluted, may be given every three or four hours.

HOFFMAN'S ANODYNE AS A MEDICINAL AGENT.

BARKSDALE, GEORGE E., Ph. G., (*Bi-monthly Bulletin*) writing of Hoffman's anodyne, says: "Years ago Hoffman's anodyne was prized as one of the most important of medicinal agents composing the physician's repertory, and even now it is meet to say something in defense of this most valuable preparation that has been degraded to a place in the materia medica today that is totally inadequate to its excellent qualities. As an anodyne and antispasmodic, no one who has ever used the official preparation can ever doubt the value of it; but in recent years so many unworthy compounds having the name of Hoffman's anodyne have been sold to confiding pharmacists for the official preparation by the makers of large quantities of ether, that the real spirit has been brought into disrepute, because physicians prescribing the preparation have been so often deceived by the spurious articles, that they have about arrived at the conclusion that the anodyne is of no account, and have, consequently, ceased to prescribe it. This has certainly displayed wisdom on the part of the physicians, for to continually prescribe a drug without its affording any effect is indeed very wrong. That most of the anodyne dispensed is of no use whatever there can be no doubt, until now it is not an easy matter to secure a sample that will even answer to the rough test of the pharmacopeia. The gradual introduction of the commercial spirit has been so stealthy that many have not yet discovered the false, yet the pharmacopeias of this country and Great Britain have observed the substituted spirit, and have published tests to identify the true; while the British pharmacopeia omitted the preparation in its last revision, owing to the varied and uncertain composition of the spirit.''

---

### FOR THE BITES OF VERMIN.

| | | |
|---|---|---|
| ℞ | Naphthol . . . . . . . . . . . . | ʒi.—ʒii. 5.00 to 10.00. |
| | Ether . . . . . . . . . . . . . . . | q. s. p. diss. |
| | Menthol . . . . . . . . . . . . . | gr. jv. xlvj. 0.75 to 250. |
| | Vaselin . . . . . . . . . . . . . | ℥iij. 90.00. |

M. Sig.—External use. —*Medical News.*

---

### FOR PERTUSSIS.

| | | |
|---|---|---|
| ℞ | Guaiacol . . . . . . . . . . . . . . . . . . . | |
| | Eucalyptol . . . . . . . . . . . . . . . . | āā ʒj. 5.00. |
| | Ol. olivæ, sterilised . . . . . . . . . . . . | ʒx. 50.00. |

M.—Inject 35 minims subcutaneously daily.

—*Central. für die Gesam. Therp.*

---

### INFECTION OF SCARLET FEVER TO SUSCEPTIBLE INDIVIDUALS.

MILLARD (*British Med. Jour.* (1966); quoted in *Phila. Med. Jour.*) shows that in 4,910 cases of scarlet fever, 158 appeared to have carried the infection to susceptible individuals even after an average isolation period of eight and a third weeks from the initial symptom. The greater proportion of the cases thus infected appeared during the first week of the return of the convalescence. The source of the infection was shared by nasal discharges, ear discharges and desquamating skin.

---

### SUSPECTED TAPE WORM.

RUEDEL (*Deut. Med. Woch.*, July 28, 1898,) reports the case of a female child of thirteen years, who was markedly anemic, of slow onset, accompanied by loss of strength and peculiar athetoid movements and spasms of the extremities. Tonic treatment failing to relieve, tapeworm was suspected, when following the administration of anthelmintics the child passed a quantity of tenia saginata and subsequently improved.

